# Minority Stress, General Stress, and Family Support: Associations With Mental Health and Quality of Life in LGBTQ+ Young Adults During the Covid‐19 Pandemic

**DOI:** 10.1111/sjop.13122

**Published:** 2025-05-08

**Authors:** Matilda Wurm, Sofia Bergbom, Guendalina Di Luigi, Veronica Della Casa, Anna Malmquist

**Affiliations:** ^1^ School of Behavioral, Social and Legal Sciences Örebro University Örebro Sweden; ^2^ Department of Behavioral Sciences and Learning Linköping University Linköping Sweden; ^3^ Department of Psychology University of Bologna Bologna Italy

**Keywords:** internalized homophobia, internalized transphobia, psychological health, sexual and gender minority, transgender and nonbinary, youth

## Abstract

By simultaneously examining minority‐related stressors and general stressors experienced by the whole population, the study's aim was to provide a more comprehensive understanding of the health and well‐being of LGBTQ+ young adults in Sweden during the Covid‐19 pandemic. This study explored differences in mental health and quality of life between subgroups of young LGBTQ+ adults. Further, it explored how distal and proximal minority stressors, as well as stress related to the Covid‐19 pandemic, and family support were associated with mental health outcomes (depression, anxiety, and quality of life) using linear regression analyses in a sample of 245 young LGBTQ+ Swedish individuals. The results showed increased mental distress in transgender and nonbinary (TNB) young adults and that minority stress influences health in LGBTQ+ young adults above and beyond the general stress of living through a pandemic. Nevertheless, different predictors were significant for different outcomes. For all outcomes, family support was highlighted as an important protective factor for LGBTQ+ young adults. Findings support the minority stress model and highlight the need for interventions aimed at reducing minority stress as well as tailored support and resources for TNB individuals during times of general high stressor load. This may include support aimed at their close families.

**Summary**

- Internalized minority stress influences LGBTQ+ adults' health above and beyond general stressors
- Family support is highlighted as an important protective factor for LGBTQ+ young adults' health
- Interventions need to focus on reducing minority stress, and giving tailored support to young LGBTQ+ adults and their families, especially during times of crisis

People worldwide were influenced by the Covid‐19 pandemic, and nations responded to the threat of the virus through various degrees of lockdown. In Sweden, most of the measures taken were voluntary. The Public Health Agency of Sweden (PHAS) aimed to avoid a collapse of the Swedish healthcare system by trying to prevent large numbers of people from contracting Covid‐19 at the same time. Swedes were encouraged to restrict social contacts, work from home, and avoid traveling (Ludvigsson 2020; PHAS 2020). During periods of the pandemic, larger gatherings were prohibited. The Government Offices of Sweden (2022) assessed that generally working with recommendations and advice was the right way to go, but that measures should have been put in place earlier to protect vulnerable groups from serious infection. In April 2020, almost all Swedes reported having changed their behavior to prevent getting infected (see Ludvigsson 2020). Hence, the pandemic affected people's possibilities of movement and general everyday life. Overall, the pandemic took a toll on citizens' well‐being, including mental health, in Sweden and across the world (McCracken et al. 2020; Samji et al. 2022).

The Covid‐19 pandemic and associated restrictions seem to have affected the mental health of certain groups more than others. For example, people with low education (Gloster et al. 2020) and members of minority groups, such as LGBTQ+ people (lesbian, gay, bisexual, transgender, queer, and other sexual and gender minorities, e.g., Pedrosa et al. 2020) were shown to be especially vulnerable. Also, the pandemic generally amplified existing challenges for young people (Public Health Agency of Sweden 2022). A critical number of youth reported more family conflicts and less time spent with peers during the pandemic, which influenced their mental health negatively (Kapetanovic et al. 2021). Hence, there are reasons to focus on vulnerable groups, such as LGBTQ+ people, in times of societal crisis while considering not only education levels and age but also potential protective factors. Also, Sweden's approach differed from other countries', but most earlier studies on the influence of Covid‐19 on LGBTQ+ people have been conducted in other countries. Thus, research from a Swedish perspective may add important information to the field.

## 1 Theoretical Framework: The Minority Stress Model

Even before the pandemic, LGBTQ+ youth were identified as a risk group for mental health complaints, such as depression and anxiety (FORTE 2018; Russell and Fish 2016). This has been explained by their higher risk of experiencing social stressors—so‐called minority stress (Brooks 1981; Meyer 2003). The understanding of minority stress as a systemic influence on mental health was first formulated by Brooks (1981) in regard to lesbian women and further popularized by the formulation of the minority stress model for sexual minorities (Meyer 2003). The minority stress model is also applicable for gender minorities (Hendricks and Testa 2012). Minority stress can take the form of exposure to discrimination, violence, and threats as well as exposure to more subtle insults, assaults, and invalidations defined as microaggressions (Nadal et al. 2010). Experiences of such stressors, often referred to as *distal minority stress*, have been shown to be relevant to understanding the social situation of Swedish young LGBTQ+ people (Lundberg et al. 2022; Tyni et al. 2024).

Besides the distal stressors, minority stress is also experienced internally, as so‐called *proximal minority stress*, for example, as internalized LGBTQ phobia, fear of rejection, and hypervigilance (Meyer 2003). Internalized LGBTQ phobia has been shown to be related to the individual's self‐esteem and self‐determination (Bockting 2015; Lefevor et al. 2019; Lev 2004) and has negative effects on mental health outcomes, including depression and anxiety (Lorenzi et al. 2015; Newcomb and Mustanski 2010; Pachankis et al. 2015; Tebbe and Moradi 2016; Walch et al. 2016; Yolaç and Meriç 2020).

The minority stress model (Meyer 2003; Hendricks and Testa 2012) highlights that distal and proximal minority‐related stressors are added to *general stressors* experienced by minorities and the majority population alike. Importantly, general stressors, such as a separation or loss of employment, are not necessarily ongoing, while minority stressors are understood as chronic, since they are connected to fairly stable societal norms. Thus, when stressors experienced due to the pandemic (e.g., a general stressor, not related to LGBTQ+ identity) are added to the distal and proximal minority stressors young LGBTQ+ adults already experience, the outcome may be especially adverse, as highlighted by Salerno et al. (2020). Since transgender and nonbinary (TNB) individuals generally experience a higher stressor load and therefore a higher risk for ill health compared to cisgender individuals (Lefevor et al. 2019), it is important to focus on the potential additional influence of gender identity when studying LGBTQ+ populations. Here, people with nonbinary identities should be included, as they have been shown to experience additional stressors compared to binary‐identified trans people (Lefevor et al. 2019; Sotardi and Watson 2019). The minority stress model also stresses the importance of acknowledging protective factors for health, such as social support. For young people, support from close family has been shown to be especially important (Ryan et al. 2010). A review focusing on family support and health outcomes in young LGBTQ+ people highlighted the importance of family support for young LGBTQ+ people's mental health but also stressed the importance of closing existing research gaps (Newcomb et al. 2019). Further, qualitative research has pointed to the particular importance of a supportive family for young LGBTQ+ adults during the Covid‐19 pandemic, when social contact was highly restricted (Malmquist et al. 2023). It can therefore be assumed that family support remains an important, yet less studied, protective factor for young adults.

## 2 The Swedish Context

Sweden is a relatively open and tolerant country, where LGBTQ+ people are protected by legal rights and high social inclusion (Hooghe and Meeusen 2013; ILGA Europe 2024). However, there are still some challenges, including the need for improved protection against hate crimes and the continued existence of conversion therapy practices (ILGA Europe 2024; Swedish Agency for Youth and Civil Society 2022). Moreover, TNB individuals in Sweden, especially young individuals, face significant barriers to legal gender recognition and health care, and there is room for improvement in terms of ensuring equal access to health care for LGBTQ+ individuals (ILGA Europe 2024; Tyni et al. 2024). In addition, Sweden is also ranked lower than the other Scandinavian countries in terms of legal gender recognition and access to health care for TNB people (ILGA Europe 2024).

A loss of social contacts and an increased dependency on close family members were common consequences of the pandemic internationally and in Sweden (Fish et al. 2020; Malmquist et al. 2022, 2023). For young LGBTQ+ people, support from other LGBTQ+ people is seen as an important protective factor (Fish et al. 2020). Belonging to a sexual and/or gender minority during a pandemic might be associated with less time spent in safe and secure contexts, such as LGBTQ+ communities, with subsequent increases in mental ill‐health. This risk may be especially pronounced for LGBTQ+ people with high‐internalized LGBTQ+ phobia. Thus, as pointed out above, the “general” (i.e., not minority‐related) stress of living through a pandemic may exacerbate the negative influence of minority stressors on health. While several earlier studies have investigated distal minority stress and some have investigated proximal stressors, there is, to the best of our knowledge, no study that has combined proximal, distal, and general stressors as well as protective factors. This is important, since professionals, such as social workers, psychologists, or other healthcare providers, need to simultaneously take distal, proximal, and general stress as well as protective factors into consideration when choosing and providing an intervention for their LGBTQ+ clients.

To address these research gaps, the purpose of this study was to investigate how the Covid‐19 pandemic affected young Swedish LGBTQ+ adults' mental health in combination with distal and proximal minority stressors as well as family support, which was here hypothesized to be a protective factor. In addition, the study aimed to explore if gender minorities were especially at risk of mental ill‐health.

The specific research questions were the following:

1. Did cisgender LGBTQ+, transgender, and nonbinary young adults differ in mental health during the Covid‐19 pandemic?
2. How were distal minority stress (discrimination, violence, humiliation), proximal minority stress (internalized LGBTQ+ phobia), and general stress (the Covid‐19 pandemic's influence), as well as protective factors (family support) associated with anxiety, depression, and quality of life in LGBTQ+ young adults?

## 3 Materials and Methods

### 3.1 Procedure

The study is part of an international project (Project Global Queerantine, Gato et al. 2021). The Swedish part of the study was approved by the Swedish Ethical Review Authority in 2020 (number 2020‐03187). Participants filled out online questionnaires in August 2020.

### 3.2 Participants

The sample consisted of 244 young adults (18–35, mean = 28 years old), identifying as LGBTQ+, living in Sweden. Participants were recruited through advertisements on LGBTQ+ websites and social media. As this is a convenient sample, information about the number of people reached by the advertisement is missing. The advertisement included brief information about the study as well as a link to the questionnaire. There was no screening involved. The participants who clicked on the link were given more information about the study, including information about how data would be stored and analyzed, that participation was voluntary, and that they could quit participation at any point. Contact information for the researchers was also provided. Before filling in the questionnaire, participants gave informed consent. At data inspection, five participants were excluded due to being older than 35 years. For more demographic details about participants, see Table 1 and further description in the Results section.

**TABLE 1:** Demographic characteristisc of participants.

Measure	Cisgender LGB		Transgender		Nonbinary		Full sample	
	*N*	%	*N*	%	*N*	%	*N*	%
Number of participants	159	65	43	17	42	17	244	100
Assigned female	139	87	27	63	37	88	203	83
Sexuality								
Heterosexual	0	0	2	5	2	5	4	2
Gay/lesbian	99	62	12	28	8	19	119	49
Bi/pansexual	53	33	22	51	23	55	98	40
Other	7	4	7	16	9	21	23	9
Education								
None or elementary	2	1	4	9	2	4	8	3
High school	27	17	15	35	15	36	57	23
University	124	80	23	54	21	50	168	69
Employment								
Employed	99	56	17	40	21	50	127	52
Unemployed	11	7	7	16	1	2	19	8
Student	48	30	16	37	17	41	81	33

### 3.3 Materials

The online survey consisted of demographic questions, questions regarding how the Covid‐19 pandemic was experienced, and scales for measuring quality of life, mental health, and stress. An English version of the survey was translated into Swedish, where forward and backward translation was performed on all scales that had not previously been translated and validated in Swedish.

### 3.4 Demographic Information

Demographic information included age, ethnicity, gender assigned at birth, gender identity, education levels, employment, relationship status, and if participants had been diagnosed with Covid‐19. In this study, age, gender identity, and education level were entered as predictors in the regression models. Gender identity and education level were here dichotomized with gender as TNB or cisgender and education level as having a university education or not.

### 3.5 Mental Health

Mental health was assessed with the short version of the Depression Anxiety and Stress Scale, DASS‐21 (Alfonsson et al. 2017; Antony et al. 1998). The DASS contains 21 items loading on three subscales: depression, anxiety, and stress, of which we used the depression and anxiety subscales. Items include “I felt like I had nothing to look forward to” (depression) and “I felt I was close to panic (anxiety)”. The psychometric properties of the DASS have been reported to be acceptable to excellent in clinical and community samples. The Cronbach's alpha in the current study was 0.93 for the depression subscale and 0.84 for the anxiety subscale.

### 3.6 Quality of Life

Quality of life was assessed with the short version of the Satisfaction with Life Scale (SWLS‐3, Kjell and Diener 2021). The three‐item scale (e.g., “In most ways my life is close to my ideal.”) has demonstrated good psychometric properties, in line with the longer five‐item version. The Cronbach's alpha in the current study was 0.89.

### 3.7 Covid‐19 Impact

The impact of the pandemic was assessed using a scale developed to measure the psychosocial effects of the Covid‐19 pandemic on LGBTQ+ individuals (Gato et al. 2021). The scale loads on three subscales: Individual impact, family climate, and social isolation. Items can be answered on a scale ranging from 0 to 10, with higher scores indicating more problems. The subscales have good psychometric properties with Cronbach's alphas of 0.74, 0.72, and 0.85, respectively. The family climate scale is developed having youth/young adults in mind who live with their families of origin, which is uncommon in Sweden. Therefore, the current study used the subscale *individual impact (3 items)* (e.g., “To what extent has the Covid‐19 pandemic affected your life?”). Cronbach's alpha in this study was 0.80 for the individual impact subscale.

### 3.8 Minority Stress

Distal minority stress was measured using three items asking if participants had been verbally or physically abused or discriminated against because they belong to a sexual and/or gender minority (e.g., “Because of my sexual and/or my gender identity I have been verbally abused.”). Items were answerable with (1) “yes” or (2) “no,” resulting in scores between 3 and 6. The items ask about distal stress on different levels, which may explain why Cronbach's alpha is fairly low in the current study with *α* = 0.60.

Internalized LGBTQ+ phobia was measured with two subscales from the Lesbian, Gay, and Bisexual Identity Scale (LGBIS; Mohr and Kendra 2011). Adaptations were made to include TNB individuals by asking about gender identity in addition to sexual identity, such as: “If I could, I would choose to be heterosexual *and/or cisgender*.” The first subscale, Identity Dissatisfaction, consists of six items concerning (dis)satisfaction with LGBT identity. The second subscale, Stigma Sensitivity, consists of three items measuring the extent to which individuals experienced anxious expectations of rejection based on their LGBT identity (e.g., “I cannot feel comfortable knowing that people judge med negatively because of my sexual orientation and/or my gender identity.”). Participants rated their responses using a 7‐point Likert scale (from 1 = *Strongly disagree* to 7 = *Strongly agree*), with higher scores indicating more problems. Cronbach's alpha for the whole scale was 0.68 in the current study.

### 3.9 Family Support

Family support was measured with the Systemic Clinical Outcome and Routine Evaluation, SCORES 15 scale, which has been shown to have good psychometric properties (Carr and Stratton 2017). It includes 15 items about participants' families (e.g., “Each of us gets listened to in our family.”), answerable on a scale between 1 and 5 with total scores between 15 and 75 and higher scores indicating better family relations. Cronbach's alpha in the current study was 0.93.

### 3.10 Analyses

IBM SPSS Statistics version 29 was used for all analyses. Before conducting the analysis, Pearson correlation coefficients were calculated to control for multicollinearity. Descriptive information on demographic variables was retrieved. The normal distribution of the data was tested and, as often is the case for measurements related to psychiatric symptoms, depression and anxiety were positively skewed, since most participants did not suffer from mental ill health. Since ANOVA is understood as a robust analysis, this is not usually seen as a problem in samples with over 200 participants. Nevertheless, since this is debated and uneven group sizes may still influence results (Field 2017), a nonparametric Kruskal–Wallis was also employed, which showed results in line with the ANOVA. We therefore feel confident reporting the results of the ANOVA. For regression analyses, the normal distribution of the outcome's residuals is of importance. QQ plots showed acceptable normal distributions. Nevertheless, since visual inspections are open for interpretation, results were further investigated by running what are considered robust regressions using the Univariate General Linear Models setup in SPSS, where robust standard errors are obtained. The results of these analyses were in line with the linear regressions, which we chose to report to be able to include standardized estimates (*β*), which are not included in the general linear model output.

Hence, we used a one‐way ANOVA to compare cisgender, transgender, and nonbinary participants on anxiety, depression, and quality of life. To investigate the impact of distal minority stress, proximal minority stress, general stress, and protective factors regressed on mental health, three linear regression models were tested. Variables were entered stepwise. In the first step, we entered demographic information (being transgender or nonbinary [TNB], having a university education, and age) and minority stressors in the form of distal stress (exposure to threats and violence) as well as proximal stress (internalized LGBTQ+ phobia). In step 2, the impact of general stress (the Covid‐19 pandemic) was added. Finally, in step 3, the predictor family support was entered, thought to work as a protective factor.

## 4 Results

### 4.1 Participants

Participant's mean age was 28 years (SD = 4.6 years). The majority (65%) identified as cisgender, followed by 18% who identified as transgender and 17% who identified as nonbinary. The majority had a university education (69%) and were employed (52%) and/or studied (33%). At the time of data collection, most (62%) were in a relationship. Table 1 gives additional information about the sample.

### 4.2 Differences Among Gender Identity Subgroups in Mental Health

One‐way analysis of variance showed a significant effect of gender identity (see Table 2). Games‐Howell post hoc tests revealed that cisgender identified young adults reported significantly lower levels of anxiety and depression and higher levels of quality of life than did both transgender young adults and nonbinary young adults. Transgender young adults and nonbinary young adults did not differ from each other on any outcome variable.

**TABLE 2:** Differences in mental health between cisgender, transgender, and nonbinary young adults.

Measure	Cisgender LGB		Transgender		Nonbinary		*F*	Df	*ɳ* ^2^
	*M*	SD	*M*	SD	*M*	SD			
Anxiety	3.27^a^	3.92	5.62^b^	5.09	5.88^b^	4.08	9.63**	2, 238	0.08
Depression	5.76^a^	5.51	10.05^b^	6.72	9.41^b^	6.34	12.60**	2, 239	0.10
Quality of life	13.72^a^	4.39	9.58^b^	4.60	10.33^b^	4.15	20.73**	2, 241	0.15

*Note:* Different superscripts indicate significant differences between groups. *ɳ* ^2^ = Eta squared, which indicates the effect size where 0.06 is considered a medium effect and < 0.14 a large effect.

** *p* < 0.001.

### 4.3 Regressions

Three linear regression models were tested with Quality of life, Depression, and Anxiety as outcome variables. For all three models, demographic variables (age, university education and being TNB) and minority stressors (distal and proximal) were entered in step 1. In step 2, general stress in the form of Covid‐19 influence was added, and, finally, in step 3, family support was entered. To interpret the results and the influence of the separate predictors, it is helpful to look at the standardized estimate (β). The standardized estimate expresses the level of change in the outcome when the predictor changes one standard deviation.

#### 4.3.1 Quality of Life

All steps in the stepwise regression were significant (step 1: *F* (5) = 15.97, *p* < 0.001; step 2: *F* (6) = 16.00, *p* < 0.001; step 3: *F* (7) = 23.40, *p* < 0.001), with step 3 explaining 42% of the variance. As can be seen in Table 3, proximal minority stress and the impact of the Covid‐19 pandemic, but also being TNB, significantly predicted a lower quality of life when all predictors were entered. Family support and having a university education were associated with a higher quality of life. Looking at the standardized estimates (*β*) we can see that family support has the largest influence, with *β* = 0.41, meaning that a change of one standard deviation in family support leads to a change of 0.41 standard deviations in Quality of Life.

**TABLE 3:** Demographic factors, stressors, and family support regressed on Quality of Life.

Model	*B* (SE)	95% CI		*β*	*p*
		Lower	Upper		
1					
Constant	16.90 (2.56)	11.83	21.93		< 0.001
Age	−0.05 (0.06)	−0.17	0.08	−0.05	0.46
University education	2.22 (0.63)	0.98	3.47	0.22	< 0.001
TNB	−2.25 (0.61)	−3.44	−1.06	−0.23	< 0.001
Distal stress	0.21 (0.29)	−0.37	0.79	0.04	0.47
Proximal stress	−0.11	−0.16	−0.06	−0.26	< 0.001
2					
Constant	20.46 (2.70)	15.13	25.78		< 0.001
Age	−0.03 (0.06)	−0.15	0.09	−0.03	0.66
University education	2.02 (0.62)	0.79	3.24	0.20	0.001
TNB	−1.95 (0.60)	−3.13	−0.77	−0.20	0.001
Distal stress	−0.05 (0.30)	−0.64	0.53	−0.01	0.86
Proximal stress	−0.12 (0.03)	−0.16	−0.06	−0.26	< 0.001
Covid impact	−0.17 (0.05)	−0.27	−0.07	−0.21	< 0.001
3					
Constant	9.40 (2.93)	3.62	15.18		0.002
Age	−0.09 (0.06)	−0.19	0.03	−0.08	0.13
University education	1.50 (0.57)	0.37	2.62	0.15	0.01
TNB	−1.39 (0.55)	−2.47	−0.30	−0.14	0.01
Distal stress	0.11 (0.27)	−0.42	0.65	0.02	0.62
Proximal stress	−0.06 (0.02)	−0.11	−0.02	−0.15	0.01
Covid impact	−0.10 (0.05)	−0.19	−0.01	−0.13	0.02
Family support	0.16 (0.02)	0.12	0.21	0.41	< 0.001

*Note:* Explained variance in model 1 = 26%, model 2 = 30%, and model 3 = 42%.

Abbreviation: TNB, transgender and nonbinary.

#### 4.3.2 Depression

All steps in the stepwise regression were significant (step 1: *F* (5) = 10.85, *p* < 0.001; step 2: *F* (6) = 10.64, *p* < 0.001; step 3: *F* (7) = 13.76, *p* < 0.001), with step 3 explaining 30% of the variance. As can be seen in Table 4, being TNB, distal, and proximal stress were initially significant predictors of depression. In step 2, Covid‐impact contributed significantly to the model, with proximal stress remaining a statistically significant predictor. In step 3, family support was the only significant predictor of depression, where higher family support is associated with lower depression, expressed as a standardized estimate of β = −0.33.

**TABLE 4:** Demographic factors, stressors, and family support regressed on depression.

Model	*B* (SE)	95% CI		*β*	*p*
		Lower	Upper		
1					
Constant	10.54 (3.49)	3.67	17.42		0.003
Age	−0.12 (0.08)	−0.28	0.05	−0.09	0.17
University education	−1.65 (0.86)	−3.34	0.04	−0.13	0.06
TNB	1.91 (0.82)	0.28	3.53	0.15	0.02
Distal stress	−0.89 (0.40)	−1.67	−0.10	−0.14	0.03
Proximal stress	0.04 (0.04)	0.03	0.17	0.19	0.004
2					
Constant	6.5 (3.73)	−0.83	13.84		0.08
Age	−0.14 (0.08)	−0.30	0.03	−0.10	0.10
University education	−1.40 (0.85)	−3.08	0.27	−0.11	0.10
TNB	1.59 (0.82)	−0.03	3.20	0.13	0.05
Distal stress	−0.58 (0.41)	−1.39	0.22	−0.09	0.15
Proximal stress	0.10 (0.03)	0.03	0.17	0.19	0.003
Covid impact	0.19 (0.07)	0.06	0.32	0.18	0.005
3					
Constant	10.54 (4.18)	9.50	25.96		< 0.001
Age	−0.07 (0.08)	−0.23	0.09	−0.05	0.40
University education	−0.90 (0.81)	−2.51	0.70	−0.07	0.27
TNB	1.09 (0.79)	−0.46	2.63	0.09	0.17
Distal stress	−0.74 (0.39)	−1.50	0.03	−0.12	0.06
Proximal stress	0.05 (0.03)	−0.02	0.12	0.10	0.13
Covid impact	0.12 (0.06)	−0.01	0.25	0.11	0.07
Family support	−0.17 (0.03)	−0.23	−0.10	−0.33	< 0.001

*Note:* Explained variance in model 1 = 19%, model 2 = 22%, and model 3 = 30%.

Abbreviation: TNB, transgender and nonbinary.

#### 4.3.3 Anxiety

All steps in the stepwise regression were significant (step 1: *F* (5) = 12.15, *p* < 0.001; step 2: *F* (6) = 11.78, *p* < 0.001; step 3: *F* (7) = 13.97, *p* < 0.001), with step 3 explaining 30% of the variance. As can be seen in Table 5, distal and proximal stressors were statistical predictors of anxiety in step 1 and remained statistically significant, together with Covid‐impact in step 2. In step 3, high distal and proximal stress was associated with higher anxiety, while high family support was associated with lower anxiety. Family support had the largest influence, expressed as a standardized estimate of *β* = −0.30.

**TABLE 5:** Demographic factors, stressors, and family support regressed on anxiety.

Model	*B* (SE)	95% CI		*β*	*p*
		Lower	Upper		
1					
Constant	7.55 (2.39)	2.84	12.27		0.002
Age	−0.07 (0.06)	−0.18	0.04	−0.08	0.23
University education	−0.32 (0.59)	−1.49	0.85	0.10	0.60
TNB	0.89 (0.57)	−0.23	2.01	−0.23	0.12
Distal stress	−1.01 (0.28)	−1.55	−0.47	−0.04	< 0.001
Proximal stress	0.09 (0.02)	0.05	0.14	0.25	< 0.001
2					
Constant	4.77 (2.55)	−0.26	9.80		0.06
Age	−0.09 (−1.50)	−0.20	0.03	−0.09	0.14
University education	−0.16 (0.59)	−1.32	1.00	−0.02	0.79
TNB	0.66 (0.56)	−0.45	1.77	0.07	0.24
Distal stress	−0.80 (0.28)	−1.35	−0.24	−0.18	0.005
Proximal stress	0.09 (0.02)	0.05	0.14	0.25	< 0.001
Covid impact	0.13 (0.05)	0.04	0.22	0.18	0.005
3					
Constant	11.97 (2.91)	6.24	17.70		< 0.001
Age	−0.04 (0.06)	−0.15	0.07	−0.05	0.43
University education	0.14 (0.57)	−0.98	1.26	0.02	0.80
TNB	0.32 (0.55)	−0.75	1.40	0.04	0.56
Distal stress	−0.91 (0.27)	−1.44	−0.37	−0.21	< 0.001
Proximal stress	0.06 (0.02)	0.01	0.11	0.16	0.01
Covid impact	0.09 (0.05)	−0.002	0.18	0.12	0.06
Family support	−0.11 (0.02)	−0.15	−0.06	−0.30	< 0.001

*Note:* Explained variance in model 1 = 21%, model 2 = 24%, and model 3 = 30%.

Abbreviation: TNB, transgender and nonbinary.

## 5 Discussion

The aim of this study was to describe and understand the mental health and quality of life in a sample of LGBTQ+ young adults during the Covid‐19 pandemic and to analyze differences depending on gender identity. Based on the minority stress model, we tested if distal stress, proximal stress, and general stress, as well as family support, were associated with three different outcomes (quality of life, depression, and anxiety). Results showed that cisgender young adults from sexual minorities scored lower on depression and anxiety and higher on quality of life than transgender and nonbinary young adults.

The results of the ANOVA are in line with earlier research showing a higher risk for negative health outcomes for gender minorities compared to cisgender people during the Covid‐19 pandemic (Vázquez et al. 2023). This has also been the case in comparison to sexual minorities. This is explained by the higher risk for gender minorities to experience minority stressors, which influence health over and above general stressors such as education level and living conditions (Meyer 2003).

Transgender and nonbinary young adults did not differ from each other on our outcome measures, which differs from previous studies (Lefevor et al. 2019; Sotardi and Watson 2019).

Some differences were detected between outcomes in the present study. When adding the impact of family support in step 3, the proximal stressor internalized LGBTQ+ phobia remained significantly associated with quality of life, while both distal and proximal stressors were associated with anxiety. For depression, the association of minority stressors was no longer significant when all factors were added into the model, and only family support remained a statistically significant predictor. This adds important information compared to studies looking at one or a few of these factors at a time. While more research may be needed to investigate the factor's influence in detail before expressing firm recommendations, professionals may want to consider these differences when meeting clients with depression, anxiety, or general low quality of life.

Overall, results from the linear regression analyses showed that proximal minority stress influenced mental health above and beyond general stressors experienced by the whole population (in this case, the influence of the Covid‐19 pandemic), even though this influence was small. For anxiety, there was also a unique significant association with distal stress. Importantly, family support had a positive influence on all three outcomes. This confirms the minority stress model (Meyer 2003), where it is stipulated that distal minority stress, proximal minority stress, and general stressors, all have their unique impact on mental health, while social support protects against ill‐health.

In line with other studies (e.g., Tebbe and Moradi 2016), proximal minority stress may be especially harmful and may be important to attend to in clinical work. On the other hand, earlier studies have shown that external stressors were experienced as less frequent during the Covid‐19 pandemic due to social isolation (Malmquist et al. 2022; Scroggs et al. 2021), which would be another way of understanding the lesser influence of distal minority stress in our study. Our results, in line with earlier studies, highlight family support as an important protective factor for LGBTQ+ young adults (Newcomb et al. 2019; Ryan et al. 2010). Also, the findings are in line with other research on depression and quality of life, where social circumstances and interpersonal relationships are suggested as major players in the development and maintenance of depression (e.g., Hames et al. 2013) and quality of life (Potter et al. 2012). Findings are also consistent with previous research providing evidence of the impact of distal and proximal stressors on mental health outcomes (Lorenzi et al. 2015; Walch et al. 2016).

In line with expectations, our results showed that general stress in the form of Covid‐19 impact made a unique contribution to outcomes in the models in step 2. This indicates that experiencing general stressors may be connected to negative outcomes, especially for people who also have high scores on chronic distal and proximal stressors. In this context the fact that Covid‐19 made a unique contribution to quality of life and mental health problems also contributes additional information regarding the Swedish context because most prior studies on LGBTQ+ mental health during the pandemic have been conducted in contexts characterized by more severe lockdowns or substantially different welfare systems (Adamson et al. 2022; Gato et al. 2021; Hong and Skiba 2025; Vázquez et al. 2023).

This study has some weaknesses. First, the data are cross‐sectional. Hence, we cannot imply causality, and the direction of the influence may work in the opposite direction or be bidirectional. In addition, we did not include heterosexual cisgender young adults in our sample and can therefore not compare our sample to that population. Further, most participants were assigned female at birth, which may influence the extent to which results can be generalized. Last, the distal minority stress experiences were measured with three dichotomous yes/no questions. This may have influenced the variability of participant's scores, which in turn may have influenced the strength of the impact of external stress in the regression models. Also, the degrees of severity of experiences may have varied between participants because the scale does not differentiate between stressors on different severity levels. Hence, future studies should attempt to measure distal stressors with more nuanced measurements. Also, several other variables may have had an influence. For example, previous studies have shown that LGBTQ+ people may have had higher levels of drinking during lockdown and may have spent more time with unsupportive families, which may also have influenced outcome variables in our study (Akré et al. 2021; Gato et al. 2021; Scroggs et al. 2021).

Nevertheless, our results make a unique contribution to the minority stress literature by simultaneously using measures for proximal, distal, and general stressors, as well as a protective factor. Also, results support earlier findings that have highlighted the impact of minority stress and the importance of family support for young LGBTQ+ populations (Ryan et al. 2010). International studies have shown that young adults living with unsupportive families were particularly unwell during the pandemic (Gato et al. 2021; Malmquist et al. 2023). In Sweden, few young adults live with their parents. Nevertheless, our results show that family support is an important protective factor for LGBTQ+ young adults living through a pandemic, as it was a protective factor in all outcomes. The mental health challenges experienced by LGBTQ+ people during the COVID‐19 pandemic have been observed across diverse geographical and cultural contexts, reinforcing the broader relevance of our findings. For instance, in a large‐scale US study, Akré et al. (2021) reported significantly elevated levels of depression, anxiety, and alcohol use among LGBTQ+ people during the pandemic; similarly, Scroggs et al. (2021) identified heightened mental health risks among LGBTQ+ emerging adults in the United States as a result of social distancing measures, which disrupted access to affirming communities. Their findings underscore the importance of social connection, especially during times of crisis, and support our emphasis on the protective role of family support in the absence of broader community engagement. Finally, Adamson et al. (2022) provided further global context through a survey of over 13,000 LGBTQ+ people across 136 countries. Their study revealed that more than half of respondents were experiencing moderate disruptions in access to care (including HIV prevention), and lack of governmental support were common themes across regions. These global patterns closely mirror our findings (particularly the elevated mental health risks for transgender and nonbinary participants) and illustrate how the COVID‐19 pandemic has amplified preexisting inequities tied to minority stress and structural marginalization.

Together, these (inter)national studies suggest that while the specific manifestations of minority stress may vary across regions, the combined burden of social stigma and pandemic‐related disruptions has had consistently negative effects on LGBTQ+ mental health worldwide. Our study contributes to this global evidence base by showing that, even in a relatively inclusive welfare state like Sweden, minority stress and a lack of proximal support (particularly from family) continue to shape the well‐being of LGBTQ+ young adults. This highlights the need for interventions that reinforce protective factors—such as family acceptance and mental health support—both during and beyond times of crisis.

Overall, our results have implications for policymakers and professionals who meet sexual and gender minorities in their professional work, such as teachers, social workers, and healthcare professionals. For all the factors studied in the current research, professionals should consider potential interventions. While distal stressors may need universal interventions on a societal level over a longer period of time, group interventions, such as in schools or in workplaces may have an influence locally. On an individual level, psychologists may consider behavioral interventions, including limiting, adding, or changing contexts that their clients frequently visit. Internalized LGBTQ+ phobia may be worked with clinically. During times of general stress, especially when these, like the pandemic, include less access to LGBTQ+ support groups and meeting places, help with finding digital groups, and other kinds of support systems may be important. Also, strengthening families of LGBTQ+ young adults should be seen as a way of giving support to this group of young adults and preventing ill health during times of crisis.

## Author Contributions

**Matilda Wurm:** main responsibility for analyses and writing of manuscript. Conceptual planning of study, together with Anna Malmquist. Preparation of data collection and responsible for data management. **Sofia Bergbom:** preliminary data analyses, writing of parts of the manuscript. **Guendalina Di Luigi:** preliminary data analyses, writing of parts of the manuscript. **Veronica Della Casa:** preliminary analyses, writing of parts of the manuscript. **Anna Malmquist:** supervision of preliminary data analyses. Conceptual planning of study, together with Matilda Wurm, writing of parts of the manuscript. All authors have been actively involved in the writing process and have read through and given input on the last version of the manuscript.

## Ethics Statement

The study was approved by the Swedish Ethical Review Authority in 2020 (number 2020‐03187).

## Conflicts of Interest

The authors declare no conflicts of interest.

## Data Availability

The data that support the findings of this study are available upon request from the corresponding author. The data are not publicly available due to privacy or ethical restrictions.

## References

[sjop13122-bib-0001] Adamson, T. , M. Hanley , S. Baral , C. Beyrer , S. Wallach , and S. Howell . 2022. “Rapid, Application‐Based Survey to Characterise the Impacts of COVID‐19 on LGBTQ+ Communities Around the World: An Observational Study.” BMJ Open 12: e041896. 10.1136/bmjopen-2020-041896. PMC9006192 35414537

[sjop13122-bib-0002] Akré, E. R. , A. Anderson , K. Stojanovski , K. W. Chung , N. A. VanKim , and D. H. Chae . 2021. “Depression, Anxiety, and Alcohol Use Among LGBTQ+ People During the COVID‐19 Pandemic.” American Journal of Public Health 111, no. 9: 1610–1619. 10.2105/AJPH.2021.306394. 34410817 PMC8589058

[sjop13122-bib-0003] Alfonsson, S. , E. Wallin , and P. Maathz . 2017. “Factor Structure and Validity of the Depression, Anxiety and Stress Scale‐21 in Swedish Translation.” Journal of Psychiatric and Mental Health Nursing 24, no. 2–3: 154–162. 10.1111/jpm.12363. 28124410

[sjop13122-bib-0004] Antony, M. M. , P. J. Bieling , B. J. Cox , M. W. Enns , and R. P. Swinson . 1998. “Psychometric Properties of the 42‐Item and 21‐Item Versions of the Depression Anxiety Stress Scales in Clinical Groups and a Community Sample.” Psychological Assessment 10, no. 2: 176–181. 10.1037/1040-3590.10.2.176.

[sjop13122-bib-0005] Bockting, W. O. 2015. “Internalized Transphobia.” In The International Encyclopaedia of Human Sexuality, edited by A. Bolin and P. Whelehan . 10.1002/9781118896877.wbiehs236.

[sjop13122-bib-0006] Brooks, V. R. 1981. Minority Stress and Lesbian Women. Lexington Books.

[sjop13122-bib-0007] Carr, A. , and P. Stratton . 2017. “The Score Family Assessment Questionnaire: A Decade of Progress.” Family Process 56, no. 2: 285–301. 10.1111/famp.12280. 28205204

[sjop13122-bib-0008] Field, A. 2017. Discovering Statistics Using SPSS Statistics. 5th ed. Sage Publications.

[sjop13122-bib-0009] Fish, J. N. , L. B. McInroy , M. S. Paceley , et al. 2020. “‘I'm Kinda Stuck at Home With Unsupportive Parents Right Now’: LGBTQ Youths' Experiences With COVID‐19 and the Importance of Online Support.” Journal of Adolescent Health 67, no. 3: 450–452. 10.1016/j.jadohealth.2020.06.002. PMC7309741 32591304

[sjop13122-bib-0010] FORTE . 2018. “Hälsa och livsvillkor bland unga HBTQ‐personer [The Health and Living Conditions of Young LGBTQ People in Sweden].” Retrieved 230428. https://forte.se/app/uploads/sites/2/2019/06/report‐young‐lgbtq‐ta.pdf.

[sjop13122-bib-0011] Gato, J. , J. Barrientos , F. Tasker , et al. 2021. “Psychosocial Effects of the COVID‐19 Pandemic and Mental Health Among LGBTQ+ Young Adults: A Cross‐Cultural Comparison Across Six Nations.” Journal of Homosexuality 68, no. 4: 612–630. 10.1080/00918369.2020.1868186. 33480823

[sjop13122-bib-0012] Gloster, A. T. , D. Lamnisos , J. Lubenko , et al. 2020. “Impact of COVID‐19 Pandemic on Mental Health: An International Study.” PLoS One 15, no. 12: e0244809. 10.1371/journal.pone.0244809. 33382859 PMC7774914

[sjop13122-bib-0013] Government Offices of Sweden . 2022. “Sverige under pandemin [Sweden During the Pandemic] SOU 2022:10.” Retrieved from Sverige under pandemin—Regeringen.se.

[sjop13122-bib-0014] Hames, J. L. , C. R. Hagan , and T. E. Joiner . 2013. “Interpersonal Processes in Depression.” Annual Review of Clinical Psychology 9: 355–377. 10.1146/annurev-clinpsy-050212-185553. 23297787

[sjop13122-bib-0015] Hendricks, M. L. , and R. J. Testa . 2012. “A Conceptual Framework for Clinical Work With Transgender and Gender Nonconforming Clients: An Adaptation of the Minority Stress Model.” Professional Psychology: Research and Practice 43, no. 5: 460–467. 10.1037/a0029597.

[sjop13122-bib-0016] Hong, C. , and B. Skiba . 2025. “Mental Health Outcomes, Associated Factors, and Coping Strategies Among LGBTQ Adolescent and Young Adults During the COVID‐19 Pandemic: A Systematic Review.” Journal of Psychiatric Research 182: 132–141. 10.1016/j.jpsychires.2024.12.037. 39809009

[sjop13122-bib-0017] Hooghe, M. , and C. Meeusen . 2013. “Is Same‐Sex Marriage Legislation Related to Attitudes Toward Homosexuality? Trends in Tolerance of Homosexuality in European Countries Between 2002 and 2010.” Sexuality Research & Social Policy: A Journal of the NSRC 10, no. 4: 258–268. 10.1007/s13178-013-0125-6.

[sjop13122-bib-0018] ILGA Europe, European Region of the International Lesbian, Gay, Bisexual, Trans and Intersex Association . 2024. “Annual Review of the Human Rights Situation of Lesbian, Gay, Bisexual, Trans and Intersex People in Europe and Central Asia.” 2024_full_annual_review.pdf.

[sjop13122-bib-0019] Kapetanovic, S. , S. Gurdal , B. Ander , and E. Sorbring . 2021. “Reported Changes in Adolescent Psychosocial Functioning During the COVID‐19 Outbreak.” Adolescents 1, no. 1: 10–20. 10.3390/adolescents1010002.

[sjop13122-bib-0020] Kjell, O. N. , and E. Diener . 2021. “Abbreviated Three‐Item Versions of the Satisfaction With Life Scale and the Harmony in Life Scale Yield as Strong Psychometric Properties as the Original Scales.” Journal of Personality Assessment 103, no. 2: 183–194. 10.1080/00223891.2020.1737093. 32167788

[sjop13122-bib-0021] Lefevor, G. T. , C. C. Boyd‐Rogers , B. M. Sprague , and R. A. Janis . 2019. “Health Disparities Between Genderqueer, Transgender, and Cisgender Individuals: An Extension of Minority Stress Theory.” Journal of Counseling Psychology 66, no. 4: 385–395. 10.1037/cou0000339. 30896208

[sjop13122-bib-0022] Lev, A. I. 2004. Transgender Emergence: Therapeutic Guidelines for Working With Gender‐Variant People and Their Families. Haworth Clinical Practice Press.

[sjop13122-bib-0023] Lorenzi, G. , M. Miscioscia , L. Ronconi , C. Pasquali , and A. Simonelli . 2015. “Internalized Stigma and Psychological Well‐Being in Gay Men and Lesbians in Italy and Belgium.” Social Science 4, no. 4: 1229–1242. 10.3390/socsci40412296.

[sjop13122-bib-0024] Ludvigsson, J. F. 2020. “The First Eight Months of Sweden's COVID‐19 Strategy and the Key Actions and Actors That Were Involved.” Acta Paediatrica 109: 2459–2471. 10.1111/apa.15582. 32951258 PMC7537539

[sjop13122-bib-0025] Lundberg, T. , A. Malmquist , and M. Wurm . 2022. “Upplevelser och hantering av minoritetsstress och mikroaggressioner bland unga hbtq‐personer i Sverige [The experience of and coping with minority stress and microaggressions among young LGBTQ people in Sweden].” In Jag är inte ensam, det finns andra som jag, edited by Myndigheten för ungdoms‐och civilsamhällesfrågor [Swedish Agency for Youth and Civil Society] . Unga hbtqi‐personers levnadsvillkor [I Am Not Alone, There Are Others Like Me. The Living Conditions of Young LGBTQI People]. Retrieved 230428. https://www.mucf.se/publikationer/jag‐ar‐inte‐ensam‐det‐finns‐andra‐som‐jag.

[sjop13122-bib-0026] Malmquist, A. , C. Bredenberg , J. Melin , M. Wurm , F. Tasker , and J. Gato . 2022. “Queers in Quarantine: Young LGBTQ+ People's Experiences During the COVID‐19 Pandemic in Sweden.” Scandinavian Journal of Psychology 64, no. 2: 150–159. 10.1111/sjop.12871. 36153699 PMC9538029

[sjop13122-bib-0027] Malmquist, A. , M. Miscioscia , D. Leal , et al. 2023. “‘Under House Arrest’: Mental Health and Minority Stress Experiences of LGBTQ+ Young Adults During the COVID‐19 Pandemic in Europe.” Sexuality Research and Social Policy 21, no. 3: 969–984. 10.1007/s13178-023-00916-x.

[sjop13122-bib-0028] McCracken, L. M. , F. Badinlou , M. Buhrman , and K. C. Brocki . 2020. “Psychological Impact of COVID‐19 in the Swedish Population: Depression, Anxiety, and Insomnia and Their Associations to Risk and Vulnerability Factors.” European Psychiatry 63, no. 1: e81. 10.1192/j.eurpsy.2020.81. 32843115 PMC7503043

[sjop13122-bib-0029] Meyer, I. H. 2003. “Prejudice, Social Stress, and Mental Health in Lesbian, Gay, and Bisexual Populations: Conceptual Issues and Research Evidence.” Psychological Bulletin 129, no. 5: 674–697. 10.1037/0033-2909.129.5.674. 12956539 PMC2072932

[sjop13122-bib-0030] Mohr, J. J. , and M. S. Kendra . 2011. “Revision and Extension of a Multidimensional Measure of Sexual Minority Identity: The Lesbian, Gay, and Bisexual Identity Scale.” Journal of Counseling Psychology 58, no. 2: 234–245. 10.1037/a0022858. 21319899

[sjop13122-bib-0031] Nadal, K. L. , D. P. Rivera , and M. J. H. Corpus . 2010. “Sexual Orientation and Transgender Microaggressions: Implications for Mental Health and Counseling.” In Microaggressions and Marginality: Manifestation, Dynamics, and Impact, 217–240. John Wiley & Sons, Inc.

[sjop13122-bib-0032] Newcomb, M. E. , M. C. LaSala , A. Bouris , et al. 2019. “The Influence of Families on LGBTQ Youth Health: A Call to Action for Innovation in Research and Intervention Development.” LGBT Health 6, no. 4: 139–145. 10.1089/lgbt.2018.0157. 30844341 PMC6551980

[sjop13122-bib-0033] Newcomb, M. E. , and B. Mustanski . 2010. “Internalized Homophobia and Internalizing Mental Health Problems: A Meta‐Analytic Review.” Clinical Psychology Review 30, no. 8: 1019–1029. 10.1016/j.cpr.2010.07.003. 20708315

[sjop13122-bib-0034] Pachankis, J. E. , S. D. Cochran , and V. M. Mays . 2015. “The Mental Health of Sexual Minority Adults in and out of the Closet: A Population‐Based Study.” Journal of Consulting and Clinical Psychology 83, no. 5: 890–901. 10.1037/ccp0000047. 26280492 PMC4573266

[sjop13122-bib-0035] Pedrosa, A. L. , L. Bitencourt , A. C. F. Fróes , et al. 2020. “Emotional, Behavioral, and Psychological Impact of the COVID‐19 Pandemic.” Frontiers in Psychology 11: 566212. 10.3389/fpsyg.2020.566212. 33117234 PMC7561666

[sjop13122-bib-0036] Potter, J. , R. Cantarero , and H. Wood . 2012. “The Multi‐Dimensional Nature of Predicting Quality of Life.” Procedia‐Social and Behavioral Sciences 50: 781–790. 10.1016/j.sbspro.2012.08.080.

[sjop13122-bib-0037] Public Health Agency of Sweden . 2020. “Ändring i föreskrifterna och allmänna råden (HSLF‐FS 20:12) om allas ansvar att förhindra smitta av covid‐19. [Change in Regulations and General Recommendations (HLSF‐FS 20:12) About Everyone's Responsibility to Prevent Infection With Covid‐19].” https://www.folkhalsomyndigheten.se/globalassets/publicerat‐material/foreskrifter/konsoliderade/hslf‐fs_2020_12.pdf.

[sjop13122-bib-0038] Public Health Agency of Sweden . 2022. “Unga och covid‐19 pandemin—ungas livsvillkor, levnadsvanor och hälsa [Young and Covid 19—Young People's Living Conditions, Living Habits, and Health].” Retrieved 241105. https://www.folkhalsomyndigheten.se/publikationer‐och‐material/publikationsarkiv/u/unga‐och‐covid‐19‐pandemin/?pub=113863.

[sjop13122-bib-0039] Russell, S. T. , and J. N. Fish . 2016. “Mental Health in Lesbian, Gay, Bisexual, and Transgender (LGBT) Youth.” Annual Review of Clinical Psychology 12: 465–487. 10.1146/annurev-clinpsy-021815-093153. PMC4887282 26772206

[sjop13122-bib-0040] Ryan, C. , S. T. Russell , D. Huebner , R. Diaz , and J. Sanchez . 2010. “Family Acceptance in Adolescence and the Health of LGBT Young Adults.” Journal of Child and Adolescent Psychiatric Nursing 23, no. 4: 205–213. 10.1111/j.1744-6171.2010.00246.x. 21073595

[sjop13122-bib-0041] Salerno, J. P. , J. Devadas , M. Pease , B. Nketia , and J. N. Fish . 2020. “Sexual and Gender Minority Stress Amid the COVID‐19 Pandemic: Implications for LGBTQ Young Persons' Mental Health and Well‐Being.” Public Health Reports 135, no. 6: 721–727. 10.1177/0033354920954511. 33026972 PMC7649999

[sjop13122-bib-0042] Samji, H. , J. Wu , A. Ladak , et al. 2022. “Review: Mental Health Impacts of the COVID‐19 Pandemic on Children and Youth—A Systematic Review.” Child and Adolescent Mental Health 27, no. 2: 173–189. 10.1111/camh.12501. 34455683 PMC8653204

[sjop13122-bib-0043] Scroggs, B. , H. A. Love , and C. Torgerson . 2021. “COVID‐19 and LGBTQ Emerging Adults: Risk in the Face of Social Distancing.” Emerging Adulthood 9, no. 5: 639–644. 10.1177/2167696820968699.

[sjop13122-bib-0044] Sotardi, V. A. , and P. W. S. J. Watson . 2019. “A Sample Validation of the Adolescent Stress Questionnaire (ASQ) in New Zealand.” Stress and Health 35, no. 1: 3–14. 30221811 10.1002/smi.2834

[sjop13122-bib-0045] Swedish Agency for Youth and Civil Society . 2022. “Jag är inte ensam, det finns andra som jag: Rapport om unga hbtqi‐personers levnadsvillkor.” Retrieved 241105. www.mucf.se/publikationer/jag‐ar‐inte‐ensam‐det‐finns‐andra‐som‐jag.

[sjop13122-bib-0046] Tebbe, E. A. , and B. Moradi . 2016. “Suicide Risk in Trans Populations: An Application of Minority Stress Theory.” Journal of Counseling Psychology 63, no. 5: 520–533. 10.1037/cou0000152. 27089059

[sjop13122-bib-0047] Tyni, K. , M. Wurm , and A. Bratt . 2024. “A Thematic Analysis of the Experiences of Prepubertal Transgender and Gender‐Diverse Children in Sweden.” Journal of LGBT Youth. 10.1080/19361653.2024.2347957.

[sjop13122-bib-0048] Vázquez, I. , J. Gato , S. Coimbra , et al. 2023. “Psychological Adjustment Profiles of LGBTQ+ Young Adults Residing With Their Parents During the COVID‐19 Pandemic: An International Study.” International Journal of Environmental Research and Public Health 20, no. 4: 3188. 10.3390/ijerph20043188. 36833881 PMC9964666

[sjop13122-bib-0049] Walch, S. E. , S. T. Ngamake , W. Bovornusvakool , and S. V. Walker . 2016. “Discrimination, Internalized Homophobia, and Concealment in Sexual Minority Physical and Mental Health.” Psychology of Sexual Orientation and Gender Diversity 3, no. 1: 37–48. 10.1037/sgd0000146.

[sjop13122-bib-0050] Yolaç, E. , and M. Meriç . 2020. “Internalized Homophobia and Depression Levels in LGBT Individuals.” Perspectives in Psychiatric Care 57, no. 1: 304–310. 10.1111/ppc.12564. 32557669

